# Unusual Clinical Presentation of Periodic Paralysis: Case Report and Literature Review

**DOI:** 10.7759/cureus.7227

**Published:** 2020-03-09

**Authors:** Abdulrahman Katabi, Pedro Ottaviano

**Affiliations:** 1 Internal Medicine, Joan C. Edwards School of Medicine, Marshall University, Huntington, USA; 2 Pulmonary Critical Care, Joan C. Edwards School of Medicine, Marshall University, Huntington, USA

**Keywords:** periodic paralysis, hypokalemia, hypokalemic periodic paralysis, muscle paralysis, stroke, muscle weakness, focal paralysis

## Abstract

We are presenting a case of hypokalemic paralysis in a patient who presented to the emergency department (ED) with a unique clinical picture that did not fully fit with other causes of periodic paralysis (hypokalemic periodic paralysis, thyrotoxic periodic paralysis, hyperkalemic periodic paralysis, and Anderson syndrome). The patient presented to the ED complaining of two days of severe flaccid paralysis in both legs and left arm; his right arm was completely normal. Initially, he was treated as a stroke alert patient and had head and spine computed tomography (CT) scans and both showed no acute pathologic changes. Initial labs showed a potassium level of 1.9 and a magnesium level of 1.8. Electrocardiography (EKG) showed prolonged QTc of 534 ms. The patient was admitted to the ICU and started on intravenous and oral potassium replacement. Over the next 24 hours, he started to regain his muscle power gradually until it came back to his baseline. Repeat EKG also showed QTc back to normal. We compared our patient's initial presentation to other published case reports with periodic paralysis and found that his initial presentation was different than other published cases.

## Introduction

Focal muscle paralysis is an alarming symptom in the emergency department (ED), particularly when it presents acutely in a young, healthy patient without predisposing factors for stroke, such as hypertension or diabetes. Periodic paralysis is a rare condition that affects muscle ion channels and may be genetic or acquired [1-2]. It can also be associated with one of four different diseases: hypokalemic periodic paralysis, thyrotoxic periodic paralysis, hyperkalemic periodic paralysis, and Anderson syndrome [2-4]. Potassium plays an important role in the physiologic functions of different tissues and membranes in the body like the heart, skeletal muscles, and nervous system. The clinical presentation in patients with hypokalemia ranges from mild fatigue and constipation to severe muscle weakness with necrosis and cardiac arrhythmias. The effect of hypokalemia symptoms depends on the severity and acuity of the change in the potassium level. [5]

## Case presentation

A 40-year-old male patient presented to the ED complaining of worsening severe bilateral leg and left arm weakness that started two days prior and was precipitated by physical exercise. His past medical history revealed hypogonadism, depression, hyperlipidemia, and cervical disc prolapse at C6-C7.

In the emergency room (ER), he was awake, alert, and oriented. The patient was in emotional distress due to his paralysis. Upon more questioning, the patient denied any history of recent fevers, chills, nausea, vomiting, diarrhea, shortness of breath, loss of consciousness, chest pain, or losing control urine or stool. Saddle area sensation was intact. The patient has no visual or hearing changes.

The patient mentioned that he had a similar episode of milder lower limb weakness a few months earlier. He linked it to intra-articular steroid injection for shoulder pain management. He did not seek medical attention for it at that time because it resolved by itself gradually.

Physical examination revealed initial vitals of blood pressure of 126/63 mmHg, heart rate 56 regular, respirations 18, and a temperature of 97.7°F (36.5°C). Flaccid paralysis was noted in the bilateral lower limbs and right arm; however, his left arm muscle power was completely normal. The sensation was intact all over. The patient had no facial numbness or weakness and had normal extraocular muscle movements. The vibration examination was intact and equal in all limbs. Deep tendon reflexes were attenuated but symmetrical in all limbs. No focal tenderness was identified on the spine. Other systems on the physical exam were within normal limits.

**Figure 1:**
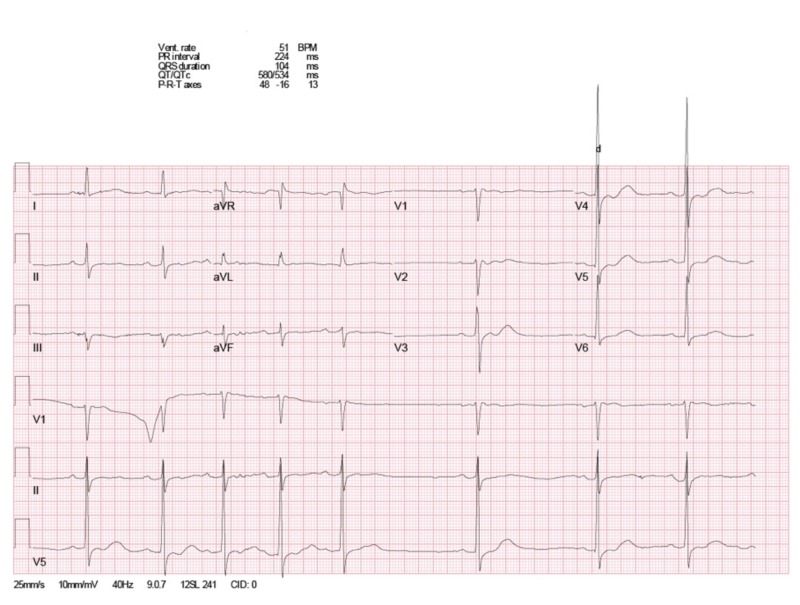
Electrocardiogram on presentation

**Figure 2:**
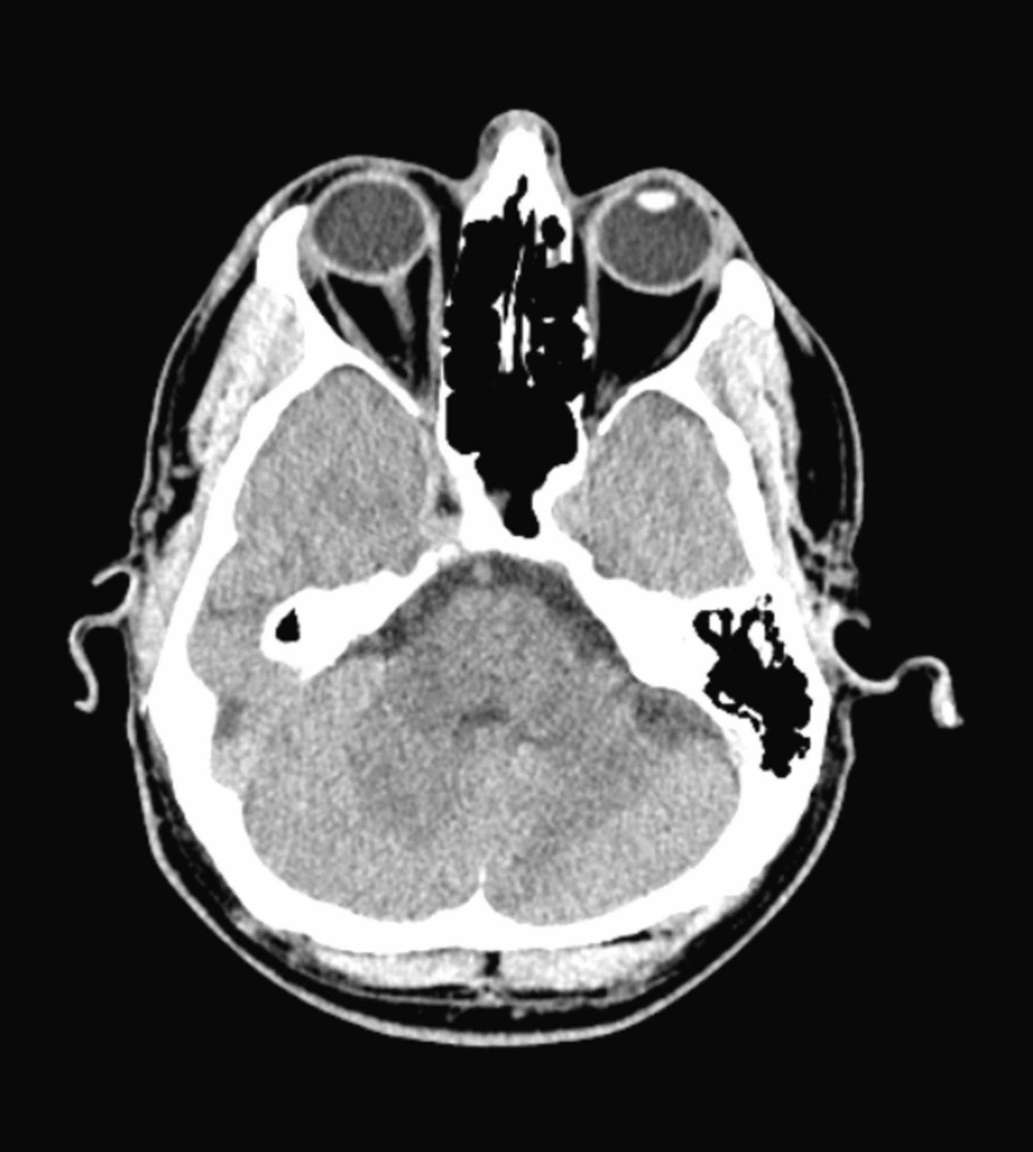
Computed tomography of the head

**Figure 3:**
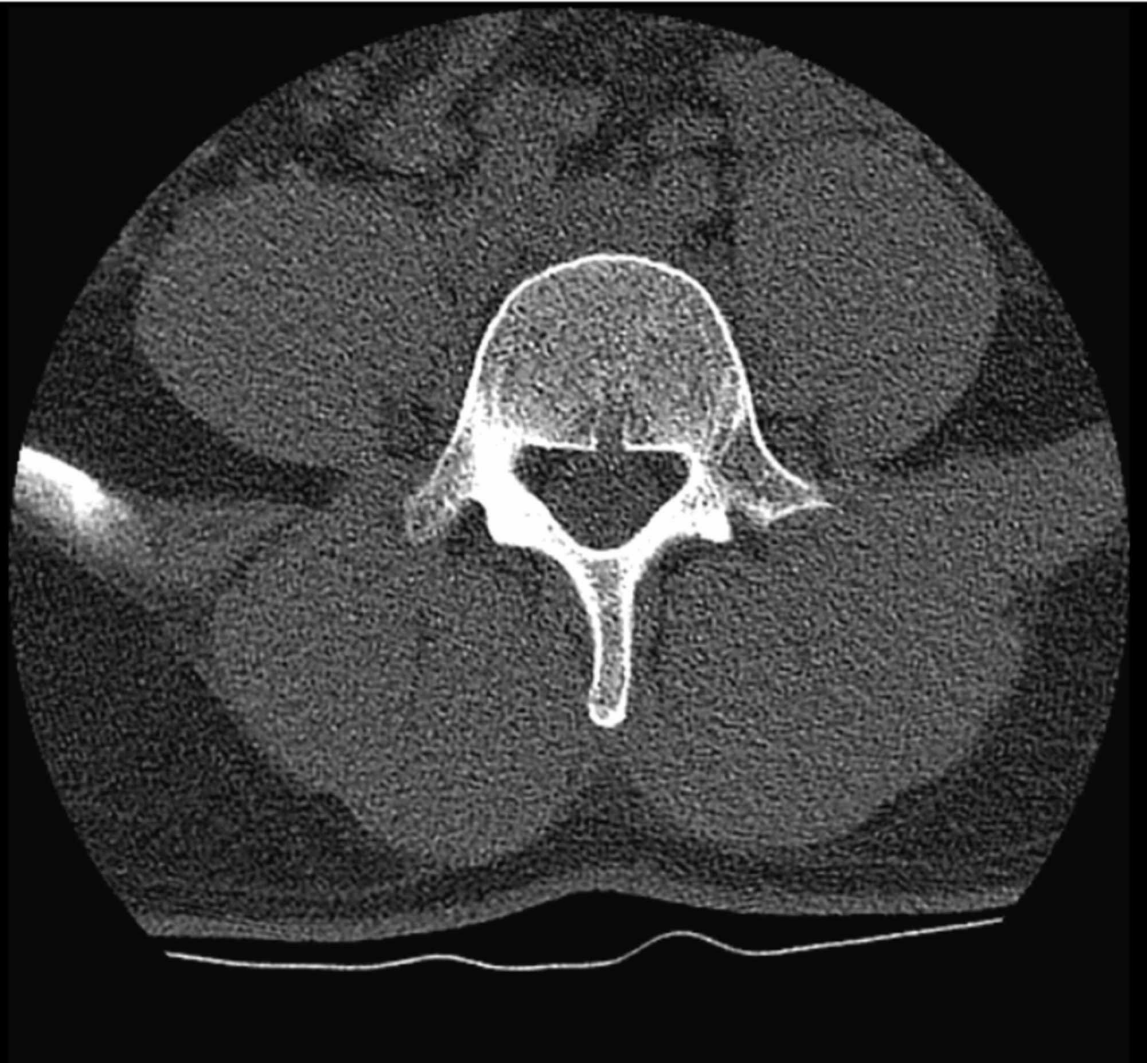
Computed tomography of the lumbar spine

Initial labs showed low serum potassium at 1.9 mmol/L (normal range: 3.5 - 5 mmol/L), minimally elevated creatine phosphokinase level at 398 IU/L (normal range: 55 - 170 IU/L), and a low normal serum magnesium level at 1.8 mg/dl (normal range: 1.8 - 2.4 mg/dl). The urine drug screen was positive for opiates (known opioid use for the patient’s chronic neck pain). Thyroid function tests were normal. Electrocardiogram (EKG) showed sinus bradycardia with a heart rate at 51 beats per minute, a long QTc interval at 534 ms, and a first-degree heart block (PR interval 224 ms) (Figure 1). Head computed tomography (CT) without contrast showed posterior scalp soft tissue swelling and mild sinus disease, but no evidence of acute intracranial hemorrhage, mass, or acute infarction (Figure 2). Lumbar CT without contrast showed multilevel mild to moderate degenerative disc disease changes, worst at the level of L4-L5 with mild to moderate broad-based disc bulging and mild narrowing of neural foramina on either side (Figure 3).

In the ED, the patient was started on intravenous (IV) potassium chloride (KCl) replacement with oral KCl as well and then admitted to the intensive care unit (ICU). In the ICU, oral KCl tablets were continued, in addition to IV magnesium sulfate (MgSO4). In the next 48 hours, the potassium level reached the upper normal level and the KCl supplementation was stopped. The patient regained his muscle power completely and he was able to walk without assistance. Repeat EKG showed QTc interval was back to baseline 394 ms. The patient then started on physical therapy and was scheduled to follow up with nephrology as an outpatient. We also advised the patient to get laboratory studies to check his electrolytes in a week. We offered him further testing in the hospital to investigate his condition, but he preferred to think about further testing after discharge.

Before discharge, we reviewed his home medication list for possible effects on electrolytes. We found that some of his medications might have a very minimal effect on lowering potassium levels. These medications included hydrocodone, fluoxetine, duloxetine, and daily testosterone transdermal patches. A formal consultation with psychiatry and endocrine specialists were performed, and they recommended to continue the psychiatric medications and to hold the testosterone patches for several weeks and observe.

## Discussion

To our knowledge, this is the first time that a patient with hypokalemic paralysis presented with paralysis in three out of four limbs. The clinical presentation of this patient was different than other types of periodic paralysis mentioned in the introduction. He had some features that overlapped with other typical presentations, but he also had other pertinent medical history and physical exam findings that made his case unique. The closest type of periodic paralysis to fit our patient would be hypokalemic periodic paralysis. This has an autosomal dominant genetic background, but our patient denied that any of his family members had the same symptoms. In comparison to thyrotoxic periodic paralysis, he did not show symptoms of hyperthyroidism and his thyroid function tests were within normal limits. Furthermore, compared to Anderson syndrome, an autosomal dominant disease that has classic dysmorphic features, our patient did not carry the dysmorphic features and none of his family members had genetic or dysmorphic diseases. Getting 24-hour urinary potassium levels is required for a definitive diagnosis, but it is challenging in acute care settings. In this situation, acute treatment and potassium replacement were preferred over urine collection for an accurate diagnosis. Also, urine collection will likely give false results due to prompt potassium replacement by the ED physicians upon presentation. In the following table, we reviewed case reports on periodic paralysis to compare the initial clinical findings in our patient to those noted in other case reports.

**Table 1:** Presenting Symptoms in Other Case Reports

Case report	Presenting symptom	Other considerations/associations
Our patient	Severe focal flaccid paralysis in all limbs, except the left upper limb.	History of recent exercise
Frappaolo 2019 [6]	Paralysis episodes from neck level down	Pregnant. History of recent exercise
Meregildo-Rodríguez 2018 [7]	Ascending paralysis	Type 1 renal tubular acidosis
Belayneh 2014 [8]	Bilateral flaccid symmetrical paralysis in arms and legs	Thyrotoxicosis
Lin 2012 [9]	Bilateral lower limbs paralysis	Thyrotoxic picture. Patient had thyroidectomy for Papillary thyroid carcinoma
Lin 2012 [9]	Generalized weakness especially in lower limbs	Thyrotoxic picture. Patient had thyroidectomy for suspicious cancer, post-surgical pathology revealed adenomatous goiter with papillary hyperplasia and lymph node with reactive hyperplasia
Gómez-Torres 2011 [10]	Lower limbs paralysis	Thyrotoxicosis
Winczewska-Wiktor 2007 [11]	Unspecified weakness	Positive family history and positive genetic abnormality present in the patient.
Erem 2005 [12]	Episodic flaccid quadriplegia in proximal muscles	Thyrotoxicosis
Seshadri 2002 [13]	Bilateral lower extremity weakness	Thyrotoxicosis
Grzesiuk 2002 [14]	Flaccid paralysis more profound in the lower limbs	Thyrotoxicosis
Ghosh 1994 [15]	Episodic weakness in the lower limbs	Tropical sprue
Gold 1992 [16]	Non-specified muscle weakness	Nocturnal attacks
Shires 1978 [17]	Profound lower limbs weakness	Beer intake before the attacks

## Conclusions

Raising awareness about focal periodic paralysis without a family history or thyroid disease can protect such patients from unnecessary radiological exposure to rule out strokes in low-risk patients. Also, it saves the health system cost of doing MRI for low-risk patients to rule out a stroke.
